# Care fragmentation and readmission mortality and length of stay before and during the COVID-19 pandemic: data from the National Readmissions Database, 2018–2020

**DOI:** 10.1186/s12913-024-11073-1

**Published:** 2024-05-14

**Authors:** Sara Turbow, Tiffany Walker, Steven Culler, Mohammed K. Ali

**Affiliations:** 1 Division of General Internal Medicine, Department of Medicine, Emory University School of Medicine, 49 Jesse Hill Jr Dr SE, Atlanta, GA 30303 USA; 2 Department of Family & Preventive Medicine, Emory University School of Medicine, Atlanta, GA USA; 3 Department of Health Policy and Management, Rollins School of Public Health, Emory University, Atlanta, GA USA; 4 Hubert Department of Global Health, Rollins School of Public Health, Emory University, Atlanta, GA USA

**Keywords:** Care fragmentation, COVID-19, In-hospital mortality, Length-of-stay

## Abstract

**Importance:**

A quarter of all 30-day readmissions involve fragmented care, where patients return to a different hospital than their original admission; these readmissions are associated with increased in-hospital mortality and longer lengths-of-stay (LOS). The stress on healthcare systems at the beginning of the COVID-19 pandemic could worsen care fragmentation and related outcomes.

**Objective:**

To compare fragmented readmissions in 2020 versus 2018–2019 and assess whether mortality and LOS in fragmented readmissions differed in the two time periods.

**Design:**

Observational study

**Setting:**

National Readmissions Database (NRD), 2018–2020

**Participants:**

All adults (> 18 y/o) with 30-day readmissions

**Main outcomes and measures:**

We examined the percentage of fragmented readmissions over 2018–2020. Using unadjusted and adjusted logistic and linear regressions, we estimated the associations between fragmented readmissions and in-hospital mortality and LOS.

**Results:**

24.0–25.7% of readmissions in 2018–2020 and 27.3%-31.0% of readmissions for COVID-19 were fragmented. 2018–2019 fragmented readmissions were associated with 18–20% higher odds of in-hospital mortality compared to nonfragmented readmissions. Fragmented readmissions for COVID-19 were associated with an 18% increase in in-hospital mortality (AOR 1.18, 95% CI 1.12, 1.24). The LOS of fragmented readmissions in March-November 2018–2019 were on average 0.81 days longer, while fragmented readmissions between March-November of 2020 were associated with a 0.88–1.03 day longer LOS.

**Conclusions and relevance:**

A key limitation is that the NRD does not contain information on several patient/hospital-level factors that may be associated with the outcomes of interest. We observed increased fragmentation during COVID-19, but its impact on in-hospital mortality and LOS remained consistent with previous years.

**Supplementary Information:**

The online version contains supplementary material available at 10.1186/s12913-024-11073-1.

## Key points

**Question:**

What was the monthly rate of fragmented readmissions during the early months of the COVID-19 pandemic (March-December 2020), and how was it different from 2018–2019? Were there differences in in-hospital mortality and LOS associated with fragmented readmissions during March-December 2020 compared with 2018–2019?

**Findings:**

In this observational study of United States data from 2018–2020, the prevalence of fragmented readmissions increased throughout 2020, but outcomes associated with fragmented readmissions were similar to those observed in 2018–2019.

**Meaning:**

While more common, fragmented readmissions were not significantly associated with increased mortality and longer length of stay observed during the first months of the COVID-19 pandemic.

**Supplementary Information:**

The online version contains supplementary material available at 10.1186/s12913-024-11073-1.

## Introduction

When a patient is readmitted to a different hospital than they were previously discharged from, interhospital fragmentation of care occurs. These fragmented readmissions account for around 25% of all 30-day readmissions [1–3] and are associated with a range of negative patient and health system outcomes, including an estimated 20% higher odds of dying during the readmission [1–5], over 75% greater odds of duplicate radiology procedures [6, 7], longer lengths-of-stay (LOS) [1, 5, 8], and over two times the odds of subsequent readmissions [9–12].

The unprecedented shifts in healthcare that occurred at the beginning of the COVID-19 pandemic in the United States created an environment ripe for fragmented care. Suspension of nonessential services, avoidance of routine or nonemergent care [13], and influxes of COVID-19 patients into hospitals and intensive care units [14–16] led to emergency departments and ICUs operating at or near capacity more frequently [17–19]. This subsequently may have increased the frequency of hospital diversion, as has been described early in the pandemic in Italy [17], leading more patients to receive care at hospitals that may not be their “home” hospital.

The effect of fragmented readmissions and the COVID-19 pandemic on patient outcomes may be negatively synergistic. Many of the risk factors for COVID-19 mortality, such as older age, multiple chronic conditions, and lower socioeconomic status are more common in patients with fragmented readmissions [1, 5]. However, while there have been numerous studies examining patient and hospital characteristics associated with readmissions, mortality, and LOS in COVID-19 admissions [16, 19–22], the impact of care fragmentation has gone almost entirely unexamined. One U.K.-based study found that COVID-19 patients who required inter-hospital ICU transfers experienced short-term deterioration in their clinical status [23].

The goal of this study was to examine the monthly rate of fragmented readmissions during March-December 2020 compared to 2018–2019 and compare in-hospital mortality and LOS associated with fragmented readmissions. We also sought to assess whether fragmented readmissions for COVID-19 were associated with increased mortality and longer LOS than nonfragmented readmissions for COVID-19 and to compare this difference to non-COVID-19 fragmented and nonfragmented readmissions.

## Methods

This study used the Agency for Healthcare Research and Quality Healthcare Cost and Utilization Project’s (HCUP) National Readmissions Database (NRD) for 2018–2020. Each year of the NRD contains a record of all nonfederal hospital discharges from thirty U.S. states within a single calendar year and includes nearly 60% of all U.S. hospitalizations; it also allows for patient tracking throughout the year across hospital admissions and readmissions. Data is compiled from HCUP’s State Inpatient Databases; states opt-in to contribute data to these; each states data is standardized and cleaned by HCUP. Additionally, it contains a complex survey weighting scheme based on discharge weights that facilitates nationally-representative estimates [24]. Linkage to outside data and linkage across years of data are not allowed.

### Patient population & variable definitions

Inpatient admissions of patients aged ≥ 18 years and readmissions occurring within 30 days were included in the analysis. Hospital-to-hospital transfers and same-day stays from 2 or more hospitals were excluded [25]. To examine fragmented readmissions, we first created admission-readmission pairs for patients. Multiple pairs were created if a patient had more than one 30-day readmission: for example, if a patient was admitted on day 1, had a second admission on day 20, and a third admission on day 45, two admission-readmission pairs would be created: admission 1 + 2 and admission 2 + 3. Admissions and readmissions could be for any reason and did not need to be diagnostically related. Readmissions were considered fragmented if the admission and readmission hospitals had different hospital identification numbers.

COVID-19 readmissions were defined as readmissions with ICD-10 code U071 in any diagnosis code position [24] that occurred between March and December of 2020. The outcomes of interest were in-hospital mortality and length of stay (LOS) of the readmission. In-hospital mortality was defined as death during the readmission. LOS was measured using the cleaned LOS variable provided by HCUP.

Because the NRD does not allow linkages across years of data, admissions in December may have 30-day readmissions in January of the following year that would not be available in the NRD, similarly, it is not possible to know if January admissions represent index admissions or readmissions from the previous year. This, coupled with the beginning of COVID in the U.S. in March of 2020 and seasonality of admissions, led us to limit our analyses to admission-readmission pairs that occurred between March and November of each year (2018, 2019, 2020).

### Analytic approach

We described the characteristics of the entire sample using weighted descriptive statistics according to the scheme provided by HCUP. Because each year has their own weights, variables across years and within years were compared using point estimates and 95% confidence intervals.

We created weighted unadjusted and adjusted logistic and linear regression models to evaluate the relationship between fragmented readmissions and the odds of in-hospital mortality and readmission LOS, clustered at the hospital level. As sub-analyses, we examined these outcomes separately for pairs where the admission had a diagnosis of COVID-19 and the readmission was for any reason, as well as pairs where both the admission and readmission were due to COVID-19.

Patient and hospital characteristics were controlled for in adjusted models. These included sex, age, zip income quartile, insurance payer, whether the patient was a resident of the state they were admitted in, the All-Patient Refined Diagnosis-Related Group (APR-DRG) risk of mortality measure, and the Elixhauser comorbidity score. Hospital characteristics included if the hospital was a teaching hospital or not, hospital urban/rural status (large metropolitan, small metropolitan, micropolitan, or “other”), and hospital control/ownership (nonfederal government, private/nonprofit, or private/invest-own). These covariates were chosen based on previous analyses of factors shown to affect odds of fragmented readmissions, in-hospital mortality, and/or LOS [1, 9]. The zip income quartile variable estimates the median household income within the patient’s zip code. The APR-DRG risk of mortality uses readmission DRGs to estimate four risk subclasses: minor, moderate, major, or extreme likelihood of dying. All hospital characteristics were measured for the readmission hospital. Additionally, for regressions examining the LOS of the readmission, the LOS of the index admission was included in adjusted models.

We completed several sensitivity analyses. First, we limited the analysis to primary COVID-19 readmissions, defined as those with ICD-10 code U071 as the primary diagnosis (first diagnosis position only) between March and November 2020. We also did the same for index admissions with a primary diagnosis of COVID-19. We then assessed the relationship between fragmented readmissions and LOS only in patients who survived their readmission, as patients who die during the readmission may have a shorter LOS. We then stratified the main analysis by the number of admission-readmission pairs each patient had: 1, 2, or > 2 to evaluate differences in patients with multiple readmissions. Finally, we completed the main analyses using 90-day readmissions rather than 30-day readmissions.

The data that support the findings of this study are available from the Healthcare Cost and Utilization Project, but restrictions apply to the availability of these data, which were used under license for the current study, and so are not publicly available. All analyses were completed in SAS 9.4 (Cary, NC) using weighted survey procedures. This study was deemed exempt from IRB review by the Emory University Institutional Review Board.

## Results

The 2018 NRD contained 17,686,511 hospital admissions. After transforming the data into admission-readmission pairs and limiting the dataset to patients ≥ 18 years old with 30-day readmissions that did not represent hospital-to-hospital transfers, 3,802,148 weighted admission-readmission pairs remained. The 2019 NRD had 18,132,865 admissions and a final weighted analytic sample of 3,837,070 admission-readmission pairs, while the 2020 NRD began with 16,692,694 admissions and led to a final weighted analytic sample of 3,436,563 admission-readmission pairs (Appendix Figure 1).

**Fig. 1:**
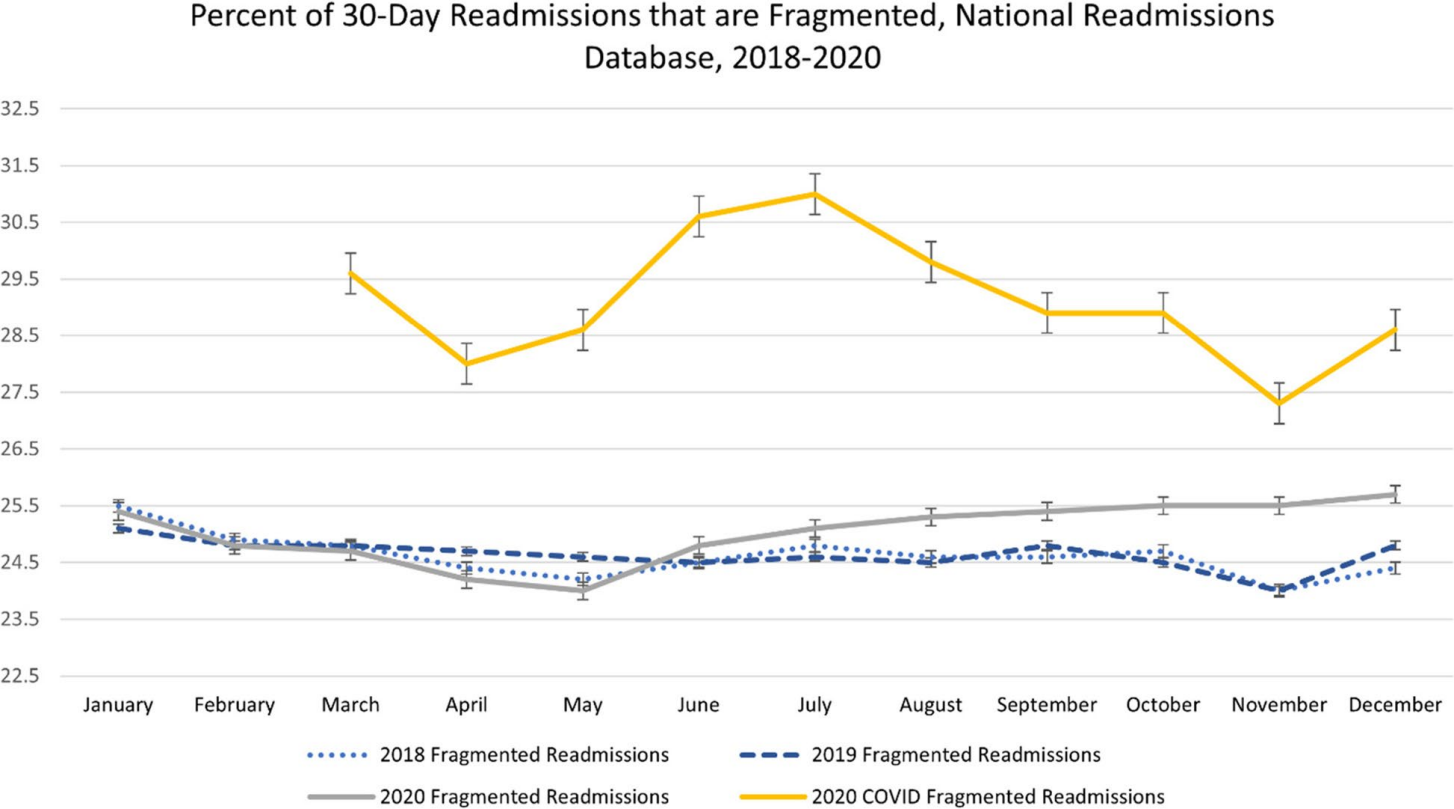
Percent of 30-day readmissions that are fragmented, National Readmissions Database, all readmissions from 2018–2020, COVID readmissions 2020. Source/notes: Author’s analysis of the National Readmissions Database, 2018–2020

From January 2018-February 2020, there were between 2.8 and 3.1 million hospital admissions each month, with a significant decline observed in March 2020 (Appendix Figure 2). Similarly, 30-day readmissions between January 2018 and February 2020 ranged between 310,000 and 360,000 each month, except for December of each year, when many 30-day readmissions occur in the following year and are not accessible in the NRD. After dropping to below 250,000 in April 2020, the number of monthly readmissions returned to approximately 300,000 for the remainder of 2020 (Appendix Figure 3). Fragmented readmissions in 2018 and 2019 ranged between 24.0% and 25.5% of all 30-day readmissions. Beginning in May 2020, the percentage of 30-day readmissions that were fragmented increased steadily to a peak of 25.7% in December 2020 (Fig. 1 and Appendix Figure 4). When readmissions with a diagnosis of COVID-19 were examined separately, the percentage of 30-day readmissions that were fragmented ranged from 27.3% to a peak of 31.0% in July 2020 (Fig. 1).

Patients with fragmented readmissions in 2020 were generally similar to patients with fragmented readmissions in 2018–2019 (Table 1). Patients with fragmented readmissions in 2020 were, however, more likely to be male (2020: 53.1%; 95% CI 52.8, 53.4 v. 2018–2019: 52.0–52.2%; 95% CI 51.7, 52.5) and were more likely to be classified as having an “extreme” likelihood of dying during their hospitalization (2020: 18.4%; 95% CI 18.1, 18.7 v. 2018–2019: 15.2%; 95% CI 14.9, 15.5). Overall, 6.0% (95% CI 5.8, 6.1) of patients with fragmented readmissions in 2020 died during their readmission, compared with 4.7% of patients (95% CI 4.6–4.9) in both 2018 and 2019 (Table 1).

**Table 1:** Weighted descriptive statistics of fragmented readmissions versus nonfragmented/same hospital readmissions, National Readmissions Database, 2018–2020

*Results are (%, 95% CI) unless otherwise noted*	**2018 (*n* = 3,802,148)**		**2019 (*n* = 3,837,070)**		**2020 (*n* = 3,436,563)**	
	**Non-fragmented**	**Fragmented**	**Non-fragmented**	**Fragmented**	**Non-fragmented**	**Fragmented**
**Male**	48.3% (48.0, 48.6)	52.0% (51.7, 52.4)	48.5% (48.3, 48.4)	52.2% (51.9, 52.5)	49.7% (49.4, 49.9)	53.1% (52.8, 53.4)
**Female**	51.7% (51.4, 52.0)	47.9% (47.6, 48.3)	51.4% (51.2, 51.7)	47.8% (47.5, 48.1)	50.3% (50.0, 50.6)	46.9% (46.6, 47.2)
**Age in years (mean, SE)**	62.1 (0.16)	59.9 (0.14)	62.2 (0.16)	60.1 (0.13)	61.7 (0.16)	60.2 (0.14)
**Zip income quartile**^**a**^						
2018: 1–45,999	31.9% (30.4, 33.4)	36.3% (35.0, 37.5)	32.5% (31.0, 34.0)	36.9% (35.6, 38.2)	32.6% (31.1, 34.1)	36.6% (35.3, 37.9)
2019: 1–47,999						
2020: 1–49,999						
2018: 46,000–58,999	28.2% (27.2, 29.2)	27.9% (27.1, 28.7)	26.5% (25.5, 27.5)	26.1% (25.4, 26.9)	28.5% (27.5, 29.5)	27.9% (27.1, 28.7)
2019: 48,000–60,999						
2020: 50,000–64,999						
2018: 59,000–78,999	23.1% (22.2, 24.0)	21.0% (20.9, 21.7)	23.6% (22.7, 24.5)	21.6% (20.9, 22.3)	21.9% (21.0, 22.7)	20.4% (19.7, 21.1)
2019: 61,000–81,999						
2020: 65,000–85,999						
2018: ≥ 79,000	16.7% (15.5, 17.9)	14.8% (13.9, 15.7)	17.4% (16.1, 18.7)	15.3% (14.4, 16.3)	16.9% (15.7, 18.2)	15.1% (14.1, 16.0)
2019: ≥ 82,000						
2020: ≥ 86,000						
**Insurance Payer**						
Medicare	59.7% (59.0, 60.4)	56.6% (56.0, 57.2)	59.4% (58.7, 60.1)	56.4% (55.9, 57.0)	57.8% (57.1, 58.5)	55.7% (55.1, 56.2)
Medicaid	17.0% (16.4, 17.7)	22.0% (21.3, 22.7)	17.2% (16.6, 17.8)	22.2% (21.6, 22.9)	18.3% (17.7, 18.8)	22.9% (22.2, 23.5)
Private	17.7% (17.1, 18.3)	14.5% (14.1, 14.9)	17.7% (17.1, 18.4)	14.5% (14.0, 14.9)	18.0% (17.4, 18.6)	14.5% (14.1, 14.9)
Self-Pay	2.8% (2.6, 3.0)	4.0% (3.7, 4.3)	2.9% (2.6, 3.1)	3.9% (3.6, 4.2)	2.9% (2.7, 3.2)	3.9% (3.6, 4.1)
No Charge	0.5% (0.4, 0.6)	0.7% (0.6, 0.9)	0.4% (0.3, 0.5)	0.6% (0.5, 0.8)	0.4% (0.3, 0.5)	0.5% (0.4, 0.7)
Other	2.2% (2.0, 2.4)	2.2% (2.0, 2.4)	2.3% (2.1, 2.5)	2.3% (2.1, 2.4)	2.6% (2.4, 2.8)	2.5% (2.3, 2.7)
**APRDRG Risk of Mortality**^**b**^						
No class specified	0.06% (0.03, 0.08)	0.06% (0.05, 0.08)	0.06% (0.03, 0.08)	0.08% (0.06, 0.09)	0.04% (0.03, 0.06)	0.05% (0.04, 0.06)
Minor likelihood of dying	23.8% (23.3, 24.3)	28.3% (27.6, 29.0)	24.9% (24.4, 25.4)	28.9% (28.2, 29.5)	23.8% (23.2, 24.3)	26.2% (25.6, 26.8)
Moderate	28.7% (28.5, 29.0)	26.9% (26.7, 27.1)	29.3% (29.0, 29.5)	27.1% (26.9, 29.5)	27.4% (27.1, 27.6)	25.2% (25.0, 25.4)
Major	33.3% (33.0, 33.7)	29.5% (29.1, 29.9)	32.3% (31.9, 32.6)	28.8% (28.4, 29.1)	32.9% (32.56, 33.2)	30.1% (29.8, 30.5)
Extreme	14.0% (13.8, 14.3)	15.2% (15.0, 15.5)	13.5% (13.3, 13.7)	15.2% (14.9, 15.4)	15.9% (15.6, 16.1)	18.4% (18.1, 18.7)
**Elixhauser Comorbidity Score**	0.68 (0.57, 0.79)	0.04 (-0.05, 0.14)	0.71 (0.59, 0.82)	-0.60 (-0.67, -0.54)	-0.34 (-0.41, -0.26)	-0.64 (-0.70, -0.57)
**Readmission Hospital Teaching Status**						
Metropolitan non-teaching	19.2% (18.1, 20.3)	23.5% (22.4, 24.6)	16.9% (15.8, 17.9)	21.0% (19.9, 22.0)	16.6% (15.5, 17.8)	20.7% (19.6, 21.8)
Metropolitan teaching	73.2% (71.9, 74.5)	66.4% (65.2, 67.7)	75.8% (74.5, 77.0)	69.1% (67.8, 70.3)	76.0% (74.7, 77.3)	69.4% (68.2, 70.7)
Non-metropolitan hospital	7.6% (6.9, 8.3)	10.0% (9.4, 10.7)	7.3% (6.6, 8.0)	9.9% (9.3, 10.6)	7.3% (6.6, 8.1)	9.9% (9.3, 10.5)
**Readmission Hospital Urban/Rural Designation**						
Large metropolitan area (> 1 million residents)	55.6% (53.2, 58.1)	60.9% (59.1, 62.7)	55.4% (52.9, 57.9)	60.1% (58.2, 62.0)	55.1% (52.5, 57.7)	60.0% (58.1, 61.9)
Small metropolitan area	36.8% (34.3, 39.1)	29.0% (27.3, 30.7)	37.2% (34.8, 39.6)	29.9% (28.2, 31.7)	37.5% (35.1, 40.0)	30.1% (28.3, 31.8)
Micropolitan	6.0% (5.3, 6.7)	6.7% (6.2, 7.3)	5.8% (5.2, 6.5)	6.7% (6.2, 7.3)	5.8% (5.1, 6.5)	6.7% (6.2, 7.2)
Non-metropolitan, non-micropolitan	1.6% (1.4, 1.8)	3.3% (3.0, 3.6)	1.5% (1.3, 1.7)	3.2% (2.9, 3.5)	1.5% (1.3, 1.7)	3.2% (2.9, 3.5)
**Readmission Hospital Control/Ownership**						
Nonfederal government	11.1% (9.4, 12.9)	11.7% (9.9, 13.4)	11.0% (9.4, 12.6)	11.5% (9.9, 13.2)	11.3% (9.7, 13.0)	11.5% (9.9, 13.0)
Private, nonprofit	75.0% (72.5, 77.5)	69.2% (66.5, 71.8)	75.3% (72.9, 77.7)	69.8% (67.3, 72.4)	75.1% (72.6, 77.5)	70.0% (67.5, 72.6)
Private, invest-own	13.8% (12.0, 15.7)	19.1% (16.9, 21.4)	13.7% (11.8, 15.5)	18.6% (16.4, 20.8)	13.6% (11.7, 15.4)	18.5% (16.3, 20.6)
**Resident of the State in which the readmission took place**						
No	4.4% (3.7, 5.1)	2.6% (2.4, 2.8)	4.5% (3.8, 5.1)	2.7% (2.5, 2.9)	4.3% (3.6, 4.9)	2.5% (2.3, 2.7)
**Admission length of stay (mean, SE)**	5.8 (0.03)	5.7 (0.03)	5.8 (0.03)	5.8 (0.03)	5.9 (0.03)	6.0 (0.03)
**Readmission length of stay (mean, SE)**	6.0 (0.03)	6.8 (0.02)	6.1 (0.02)	6.9 (0.02)	6.2 (0.03)	7.1 (0.03)
**Died during the readmission**	4.1% (4.0, 4.2)	4.7% (4.6, 4.8)	4.1% (4.0, 4.2)	4.7% (4.6, 4.9)	4.9% (4.8, 5.0)	6.0% (5.8, 6.1)

Source/notes: Author’s analysis of the National Readmissions Database, 2018–2020

^a^Updated annually, so these vary by year. Numbers for each year are listed

^b^All Patient Refined DRGs (APR-DRGs) uses the diagnosis-related group (DRG) of the admission/readmission to estimate the risk of mortality from that DRG, then groups it into one of four subclasses: minor, moderate, major, or extreme likelihood of dying

Compared to nonfragmented readmissions for COVID-19, patients with fragmented readmissions for COVID-19 were more likely to be in the lowest zip income quartile (fragmented 38.1% v. nonfragmented 34.5%, *p* < 0.0001), more likely to have Medicaid (14.6% v. 12.3%, *p* < 0.0001), and more likely to be classified as having an “extreme” likelihood of dying during their hospitalization (44.3% v. 40.9%, *p* = 0.0003) (Table 2). Overall, 20.4% of patients with a fragmented readmission for COVID-19 died during the hospitalization, compared to 17.7% with a nonfragmented/same hospital COVID-19 readmission (*p* < 0.0001). Fragmented COVID-19 readmissions had an average LOS of 10.0 days, while nonfragmented/same hospital readmissions for COVID-19 had an average LOS of 8.8 days (*p* =< 0.0001) (Table 2).

**Table 2:** Descriptive statistics of fragmented v. nonfragmented readmissions for COVID-19, National Readmissions Database, 2020

	**All 2020 Readmissions**	**Nonfragmented Readmissions for COVID-19**	**Fragmented Readmissions for COVID-19**	***P***
**Male**	50.5%	52.9%	54.6%	< 0.0001
**Female**	49.5%	47.1%	45.4%	
**Age in years** **Mean (SE)**	59.6 (0.25)	67.9 (0.1)	67.2 (0.1)	
**Zip income quartile**				
1–49,999	33.6%	34.5%	38.1%	< 0.0001
50,000–64,999	28.4%	28.5%	28.0%	
65,000–85,999	21.5%	21.5%	20.1%	
> 86,000	16.5%	15.5%	13.8%	
**Insurance Payer**				
Medicare	57.2%	70.0%	67.7%	< 0.0001
Medicaid	19.4%	12.3%	14.6%	
Private	17.1%	14.0%	12.9%	
Self-Pay	3.2%	1.9%	1.9%	
No Charge	0.4%	0.2%	0.1%	
Other	2.5%	2.6%	2.7%	
**APRDRG risk mortality**^**a**^				
No class specified	0.04%	0.01%	0.02%	< 0.0001
Minor likelihood of dying	24.4%	0.2%	0.3%	
Moderate	26.8%	10.3%	9.9%	
Major	32.2%	48.5%	45.3%	
Extreme	16.5%	40.9%	44.3%	
**Readmission Hospital Teaching Status**				
Metropolitan non-teaching	17.7%	18.0%	20.7%	< 0.0001
Metropolitan teaching	74.3%	72.5%	66.5%	
Non-metropolitan hospital	8.0%	9.5%	12.7%	
**Readmission Hospital Urban/Rural Designation**				
Large metropolitan area (> 1 million residents)	56.3%	54.5%	59.9%	< 0.0001
Small metropolitan area	35.7%	35.9%	27.3%	
Micropolitan	6.1%	7.2%	8.3%	
Non-metropolitan, non-micropolitan	1.9%	2.3%	4.4%	
**Readmission Hospital Control/Ownership**				
Nonfederal government	11.4%	11.6%	12.2%	< 0.0001
Private, nonprofit	73.8%	74.3%	67.4%	
Private, invest-own	14.8%	14.2%	20.4%	
**Resident of the State in which the readmission took place**				
No	3.8%	3.2%	2.2%	< 0.0001
**Admission length of stay (mean, SE)**	6.1 (0.04)	6.1 (0.05)	6.7 (0.06)	< 0.0001
**Readmission length of stay (mean, SE)**	6.1 (0.02)	8.8 (0.07)	10.0 (0.07)	< 0.0001
**Died during the readmission**	5.2%	17.7%	20.4%	< 0.0001

Source/notes: Author’s analysis of the National Readmissions Database, 2018-2020

^a^All Patient Refined DRGs (APR-DRGs) uses the diagnosis-related group (DRG) of the admission/readmission to estimate the risk of mortality from that DRG, then groups it into one of four subclasses: minor, moderate, major, or extreme likelihood of dying

Fragmented readmissions in March-November 2018–2019 for any reason were associated with 18–20% higher odds of in-hospital mortality in models adjusting for patient and hospital characteristics (2018 AOR 1.18, 95% CI 1.14, 1.22; 2019 AOR 1.20, 95% CI 1.12, 1.24) compared to nonfragmented/same hospital readmissions (Table 3). Fragmented readmissions for COVID-19 were associated with 18% higher in-hospital mortality compared to nonfragmented/ same hospital readmissions for COVID-19 (AOR 1.18, 95% CI 1.12, 1.24). Fragmented readmissions during the pandemic for diagnoses other than COVID-19 were associated with 17% higher odds of in-hospital mortality (AOR 1.17, 95% CI 1.13, 1.20) (Table 3).

**Table 3:** Association between fragmented readmissions and the odds of readmission in-hospital mortality, National Readmissions Database, March-November 2018, 2019, and 2020

*Odds Ratio (95% CI)*	**Unadjusted**	**Clinical/Demo**^**a**^	**Hospital**^**b**^	**Full**^**c**^
**2018**	1.14 (1.11, 1.18)	1.18 (1.14, 1.22)	1.15 (1.12, 1.19)	1.18 (1.14, 1.22)
**2019**	1.17 (1.14, 1.21)	1.20 (1.16, 1.24)	1.18 (1.15, 1.22)	1.20 (1.16, 1.24)
**2020 COVID-19 Readmissions**	1.21 (1.15, 1.26)	1.19 (1.13, 1.25)	1.20 (1.14, 1.25)	1.18 (1.12, 1.24)
**2020 Non-COVID-19 Readmissions**	1.22 (1.18, 1.26)	1.17 (1.13, 1.21)	1.22 (1.19, 1.26)	1.17 (1.13, 1.20)

Source/notes: Author’s analysis of the National Readmissions Database, 2018-2020

Reference group is nonfragmented readmissions

^a^Adjusted for sex, age, zip income quartile, insurance payer, resident of the state the readmission occurred in, APRDRG risk of mortality

^b^Adjusted for readmission hospital teaching status, readmission hospital urban/rural status, readmission hospital ownership

^c^Models a + b covariates included

The LOS of fragmented readmissions in 2018 and 2019 were an average of 0.81 days longer (95% CI 0.73, 0.89) than nonfragmented readmissions after adjusting for patient characteristics, hospital characteristics, and LOS of the index admission (Table 4). Fragmented readmissions for COVID-19 were 1.23 days longer in unadjusted models (95% CI 1.02, 1.43); after adjusting for both patient and hospital characteristics, fragmented COVID-19 readmissions were associated with a 1.03-day longer LOS (95% CI 0.83, 1.23). Fragmented readmissions in 2020 for reasons other than COVID-19 were associated with an increased LOS of 0.88 days compared to nonfragmented readmissions (95% CI 0.80, 1.96) after adjusting for patient and hospital characteristics (Table 4).

**Table 4:** Association between fragmented readmissions and the length of stay of the readmission, National Readmissions Database, March-November 2018, 2019, and 2020

*Regression coefficient (95% CI)*	**Unadjusted**	**Clinical/Demo**^**a**^	**Hospital**^**b**^	**Full**^**c**^
**2018**	0.84 (0.76, 0.91)	0.80 (0.73, 0.88)	0.86 (0.79, 0.94)	0.81 (0.74, 0.89)
**2019**	0.86 (0.77, 0.94)	0.80 (0.72, 0.88)	0.88 (0.80, 0.96)	0.81 (0.73, 0.89)
**2020 COVID-19 Readmissions**	1.23 (1.02, 1.43)	1.05 (0.84, 1.25)	1.23 (1.02, 1.43)	1.03 (0.83, 1.23)
**2020 Non-COVID-19 Readmissions**	0.96 (0.88, 1.04)	0.87 (0.78, 0.95)	0.99 (0.91, 1.07)	0.88 (0.80, 0.96)

Source/notes: Author’s analysis of the National Readmissions Database, 2018-2020

Reference group is nonfragmented readmissions

^a^Adjusted for sex, age, zip income quartile, insurance payer, resident of the state the readmission occurred in, APRDRG risk of mortality, Elixhauser Comorbidity Score, LOS of admission

^b^Adjusted for readmission hospital teaching status, readmission hospital urban/rural status, readmission hospital ownership

^c^Models a + b covariates included

In the analysis where index admissions for COVID-19 were examined, a fragmented readmission was associated with a 22% higher odds of dying during the readmission in the fully adjusted model (AOR 1.22, 95% CI 1.14, 1.30), and a 1.17-day longer LOS (95% CI 0.99, 1.35) (Appendix Table 3). In the sub-analysis limited to admission-readmission pairs in which both the admission and readmission had a diagnosis of COVID-19, a fragmented readmission was associated with 25% higher odds of dying during the readmission in the fully adjusted model (AOR 1.25, 95% CI 1.16, 1.35). Fragmented readmissions were associated with a 1.31-day longer LOS (95% CI 1.09, 1.54) after adjusting for patient and hospital characteristics (Appendix Table 4).

In sensitivity analyses limited to admissions or readmissions where COVID-19 was the primary diagnosis, the results were similar (Appendix Tables 5 & 6). When patients who died during their readmission were removed, the LOS remained 1.30 days longer in fragmented compared to nonfragmented COVID-19 readmissions (95% CI 1.09, 1.53) (Appendix Table 7). Next, when the analysis was stratified by the number of admission-readmission pairs each unique patient had in the dataset, the results for in-hospital mortality and readmission LOS were also similar to the main analysis (Appendix Tables 8 & 9). Finally, when 90-day readmissions were examined, the odds of in-hospital mortality in COVID-19 readmissions remained higher by 15% (AOR 1.15, 95% CI 1.10, 1.20) and LOS remained 0.85 days longer (95% CI 0.68, 1.02) (Appendix Tables 10 & 11) compared to nonfragmented readmissions.

## Discussion

This nationally representative study of 30-day readmissions between 2018–2020 found that while interhospital fragmentation of care increased during the COVID-19 pandemic, the effects of a fragmented readmission on in-hospital mortality were similar to pre-pandemic years. Interestingly, fragmented readmissions for COVID-19 infections between March and November of 2020 were associated with an increased LOS of over a day, a number that increased further to 1.31 days when both the admission and readmission were for COVID-19.

Notably, the rate of fragmented readmissions did not immediately increase in March 2020, when the pandemic began in earnest in the United States. From March until May 2020, the percentage of readmissions that were fragmented was similar to what was seen in prior years (Fig. 1). This likely reflects the early “anticipatory” phase of the pandemic—hospitals were not yet overflowing with COVID-19 patients and many other patients were avoiding routine care. At the end of March 2020, the COVID-19-associated hospitalization rate was 7.6 per 100,000 people [13]—by the end of December 2020, it had increased to 19.1 per 100,000 people [26]. As shown in Appendix Figure 2, overall hospitalizations decreased dramatically between March and June 2020 and rebounded somewhat in July 2020, although they had not reached pre-pandemic levels by the end of 2020. The month-by-month examination of fragmented readmissions is a strength of this analysis, as it accounts for seasonal changes in readmissions and fragmented readmissions. The increase in fragmented readmissions, particularly those for COVID-19, may be due to several factors. The first is changes in ICU capacity, as patients who required hospitalization for COVID-19 in 2020 were often very ill, and ICU capacity was often a limiting factor in a hospital’s ability to admit a COVID-19 patient [19, 20, 27, 28]. This is supported by our finding that patients with fragmented readmissions in 2020 were more likely to have an “extreme” risk of mortality compared to patients admitted in 2018 or 2019. Second, readmission rates in COVID-19 patients are very high [21, 22]. The high volume of critically ill patients with many readmissions may have forced hospitals to divert patients elsewhere, resulting in increased fragmented readmissions for both COVID-19 and non-COVID-19 patients.

Fragmented readmissions had a similar impact on in-hospital mortality during the readmission during the early months of COVID-19 as they did in 2018 and 2019 (Table 3). In the early months of the pandemic, care for hospitalized patients with COVID-19 was generally supportive and may have been relatively homogenous across hospitals, which may have decreased the impact of fragmented readmissions on the odds of dying during the readmission.

The increase in LOS associated with fragmented readmissions between March and November of 2020 was similar to what was observed for fragmented readmissions in 2018 and 2019. As above, this may have been driven by patient clinical and demographic characteristics for both COVID-19 and non-COVID-19 readmissions. However, a significantly longer readmission LOS was observed for patients whose index admission was for COVID-19 and who had a subsequent fragmented readmission also for COVID-19. These patients may have had more severe COVID-19 or experienced significant sequelae from their initial infection, which may impact their quality of care and LOS. Previous work has shown that LOS is largely driven by medical diagnoses and social determinants of health [29, 30], perhaps more so than by the care coordination challenges presented by a fragmented readmission.

Our study has several limitations. First, the NRD is limited in the patient- and hospital-level data it contains. While it is a powerful tool to track admissions and readmissions, limitations on data linkage to outside sources prevent a more detailed examination of factors such as race/ethnicity, social determinants of health, hospital markets, ambulance use [31], hospital specialization, and hospital course (i.e., intensive care unit use) that may be associated with the outcomes of interest. One key limitation of the data is that because we cannot link across years of data, we removed December admissions as many 30-day readmissions would occur in January of the following year, and because of the timing of the COVID-19 pandemic, we removed January and February admissions, which may lead to selection bias. Of particular importance to this analysis is the lack of data on patient/hospital location, as the COVID-19 pandemic did not affect all parts of the country uniformly throughout 2020. However, we adjusted for available variables at both the patient- and hospital-level. Additionally, we are not able to discern “appropriate” versus “inappropriate” care fragmentation (e.g., a fragmented readmission for a cerebrovascular accident to a stroke center might be an example of an “appropriate” instance of care fragmentation). Finally, we do not know if the “index” admission hospital is the patient’s “home” hospital.

One unmeasured consideration that may have impacted hospital LOS during the COVID-19 pandemic was the use of “field hospitals” or other nontraditional settings of care to reduce hospital burden. In Atlanta, for example, patients who were stable but not yet ready for home could be transferred to a field hospital at the Georgia World Congress Center [32], thus reducing hospital LOS. These admissions, however, are not included in the NRD. These types of novel settings of care may have been more common in urban locations, where fragmented readmissions may also be more common due to more availability of hospitals that are closer to each other [33, 34].

This study is the first to quantify fragmented readmissions in the early months of the COVID-19 pandemic. Previous work has established that fragmentation does not impact all patients the same [1, 9], here we describe how fragmented readmissions affected two important outcomes in COVID-19 hospitalizations—mortality and LOS. Future work should examine both patient- and system-level factors associated with poor patient outcomes in fragmented readmissions due to COVID-19, and should examine whether improved information sharing between hospitals, such as via health information exchanges, could improve outcomes in fragmented readmissions in public health emergencies.

### Supplementary Information

Supplementary Material 1.

## Data Availability

The data that support the findings of this study are available from the Healthcare Cost and Utilization Project, but restrictions apply to the availability of these data, which were used under license for the current study, and so are not publicly available. Data are however available from the authors upon reasonable request and with permission of the Healthcare Cost and Utilization Project.
