# Supplementary material for: Care fragmentation and readmission mortality and length of stay before and during the COVID-19 pandemic: data from the National Readmissions Database, 2018–2020
Source: BMC Health Serv Res. 2024 May 14;24:622. doi: 10.1186/s12913-024-11073-1 (PMC11090805; doi:10.1186/s12913-024-11073-1)
Supplement: Supplementary file 1 — Supplementary Material 1. [file 12913_2024_11073_MOESM1_ESM.docx]

***Appendix***

**Appendix Figure 1: Data Cleaning, National Readmissions Database 2018-2020, Unweighted**


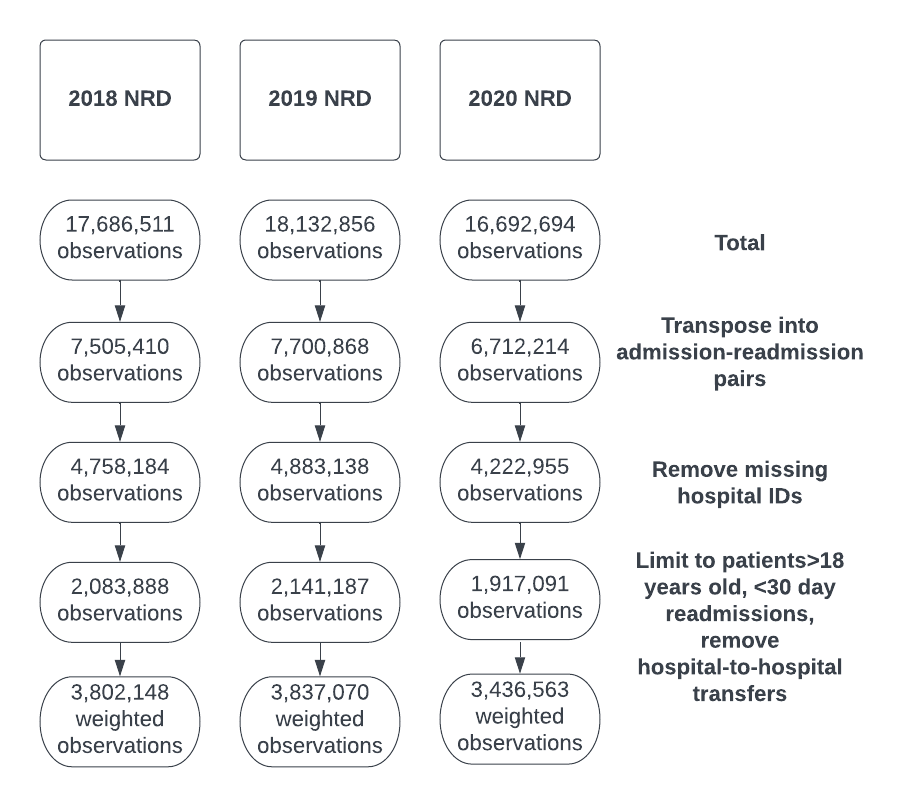


**Appendix Figure 2: Number of Hospital Admissions by Month, National Readmissions Database, 2018-2020**


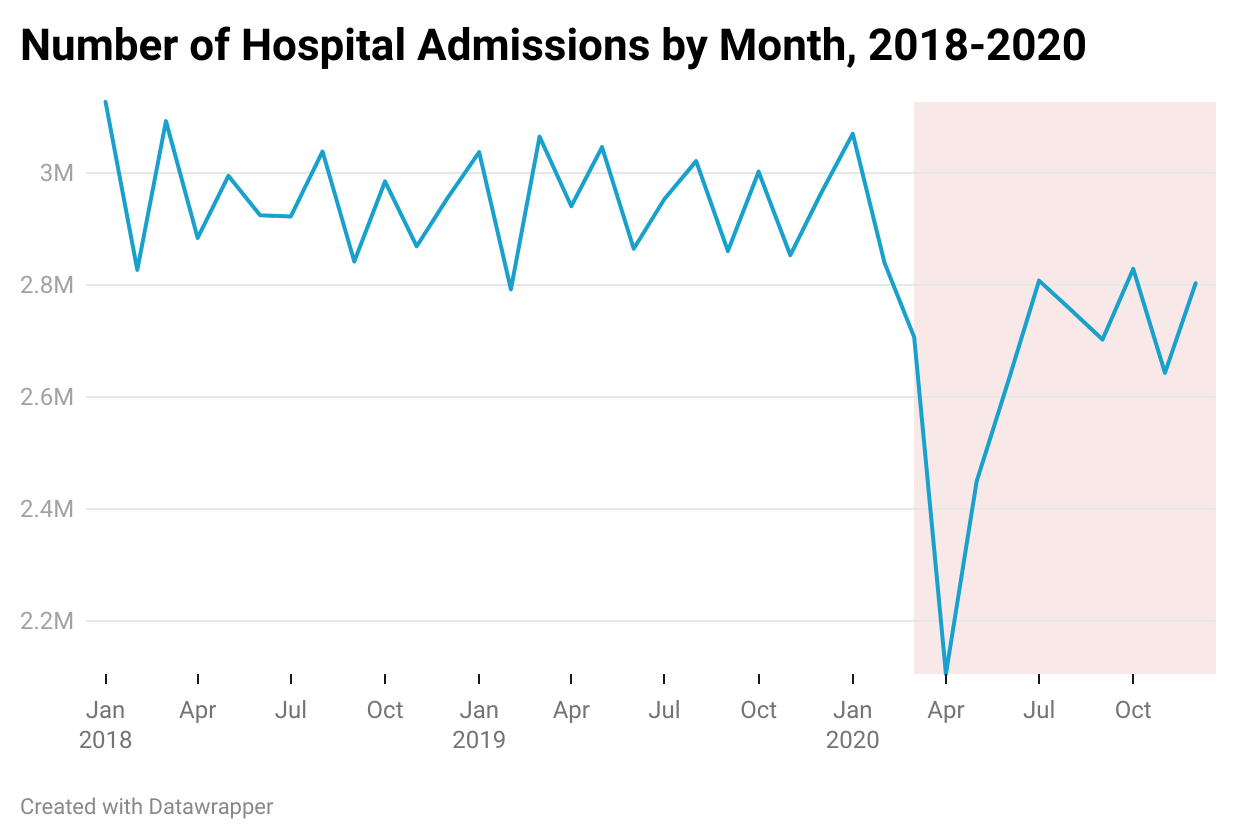


**Appendix Figure 3: Number of 30-Day Readmissions by Month, National Readmissions Database, 2018-2020**


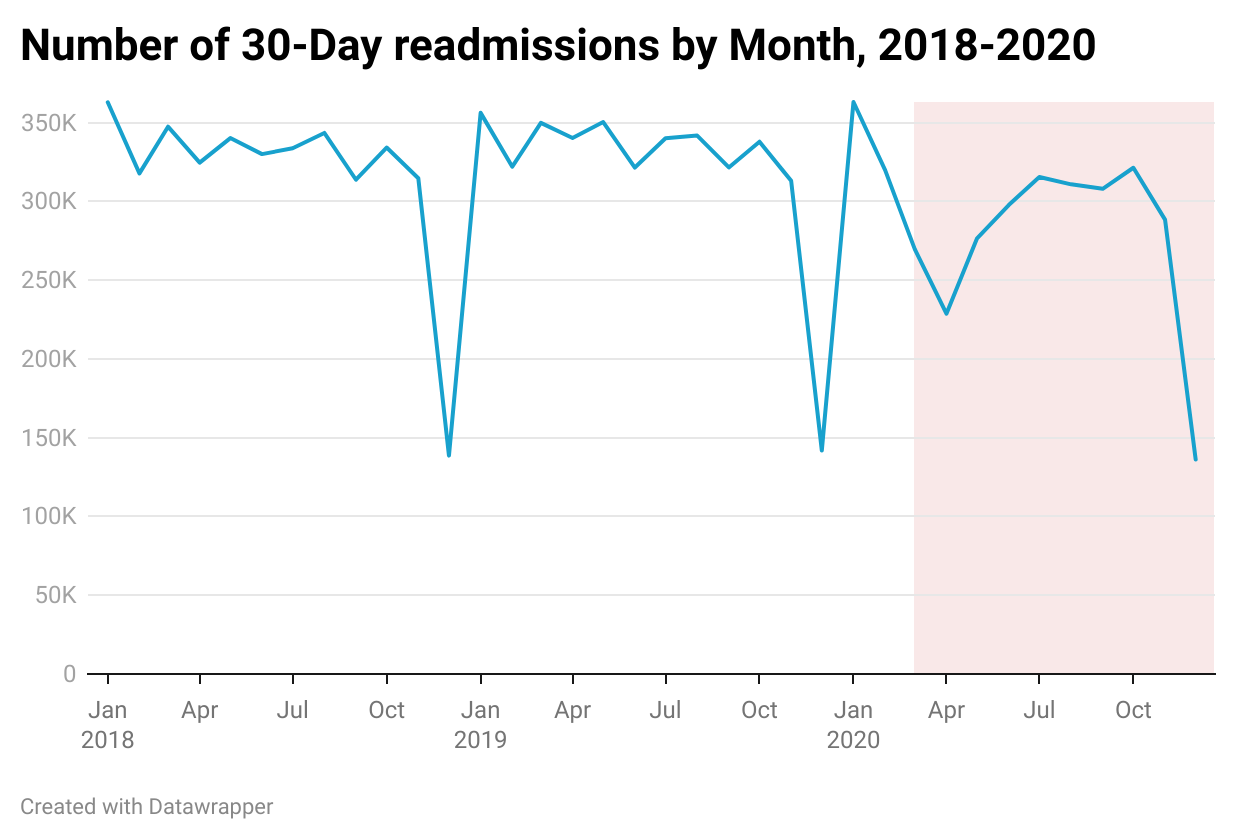


**Appendix Figure 4: Percent of 30-Day Readmissions that are Fragmented, National Readmissions Database, 2018-2020**

**Appendix Table 1: Descriptive Statistics of Fragmented v. Nonfragmented Readmissions for a Primary Diagnosis of COVID-19, 2020**

|  |  | 2020 ALL | Nonfragmented-  Primary Diagnosis of COVID-19 | Fragmented-  Primary Diagnosis of COVID-19 | p |
| --- | --- | --- | --- | --- | --- |
| Male |  | 50.5% | 53.4% | 54.4% | 0.0645 |
| Female |  | 49.5% | 46.6% | 45.6% |  |
| Age in years  Mean (SE) |  | 59.6 (0.25) | 69.8 90.14) | 68.6 (0.18) | <0.0001 |
| Zip income quartile | 1-49,999 | 33.6% | 34.8% | 39.5% | <0.0001 |
|  | 50,000-64,999 | 28.4% | 29.0% | 27.9% |  |
|  | 65,000-85,999 | 21.5% | 21.4% | 19.7% |  |
|  | ≥ 86,000 | 16.5% | 14.9% | 12.9% |  |
| Insurance Payer | Medicare | 57.2% | 71.7% | 69.4% | <0.0001 |
|  | Medicaid | 19.4% | 9.5% | 12.0% |  |
|  | Private | 17.1% | 13.9% | 13.7% |  |
|  | Self-Pay | 3.2% | 1.9% | 1.9% |  |
|  | No Charge | 0.4% | 0.2% | 0.1% |  |
|  | Other | 2.5% | 2.7% | 2.8% |  |
| APRDRG risk of mortality* | No class specified | 0.04% | 0.01% | 0.01% | 0.0003 |
|  | Minor likelihood of dying | 24.4% | 0.06% | 0.03% |  |
|  | Moderate | 26.8% | 10.3% | 10.9% |  |
|  | Major | 32.2% | 55.3% | 52.7% |  |
|  | Extreme | 16.5% | 34.3% | 36.3% |  |
| Elixhauser Comorbidity score |  | 0.034 | 0.34 | -0.04 | <0.0001 |
| Readmission Hospital Teaching Status | Metropolitan non-teaching | 17.7% | 18.8% | 19.9% | <0.0001 |
|  | Metropolitan teaching | 74.3% | 69.6% | 65.7% |  |
|  | Non-metropolitan hospital | 8.0% | 11.5% | 14.3% |  |
| Readmission Hospital Urban/Rural Designation | Large metropolitan area (>1 million residents) | 56.3% | 51.7% | 57.8% | <0.0001 |
|  | Small metropolitan area | 35.7% | 36.7% | 27.9% |  |
|  | Micropolitan | 6.1% | 8.5% | 9.3% |  |
|  | Non-metropolitan, non-micropolitan | 1.9% | 3.0% | 5.0% |  |
| Readmission Hospital Control/  Ownership | Nonfederal government | 11.4% | 12.2% | 13.0% | <0.0001 |
|  | Private, nonprofit | 73.8% | 73.8% | 66.1% |  |
|  | Private, invest-own | 14.8% | 14.0% | 20.8% |  |
| Resident of the State in which the readmission took place | No | 3.8% | 3.3% | 2.0% | <0.0001 |
| Admission length of stay (mean, SE) |  | 6.1 (0.04) | 5.6 (0.05) | 6.2 (0.08) | <0.0001 |
| Readmission length of stay (mean, SE) |  | 6.1 (0.02) | 8.3 (0.08) | 9.2 (0.09) | <0.0001 |
| Died during the readmission |  | 5.2% | 17.0% | 18.5% | 0.0009 |

** All Patient Refined DRGs (APR-DRGs) uses the diagnosis-related group (DRG) of the admission/readmission to estimate the risk of mortality from that DRG, then groups it into one of four subclasses: minor, moderate, major, or extreme likelihood of dying.*

**Appendix Table 3: Analysis of COVID-19 Admissions, National Readmissions Database, March-November 2020**

|  | Unadjusted | Clinical/Demo^a^ | Hospital^b^ | Full^c^ |
| --- | --- | --- | --- | --- |
| Died during Readmission (OR, 95% CI) | 1.29 (1.22, 1.37) | 1.24 (1.16, 1.32) | 1.28 (1.21, 1.36) | 1.22 (1.14, 1.30) |
| LOS readmission (Regression coefficient, 95% CI) | 1.45 (1.27, 1.64) | 1.18 (1.00, 1.35) | 1.44 (1.26, 1.63) | 1.17 (0.99, 1.35) |

*Reference group is nonfragmented readmissions.*

*a. Adjusted for sex, age, zip income quartile, insurance payer, resident of the state the readmission occurred in, APRDRG risk of mortality, Elixhauser Comorbidity Score, LOS of admission (LOS outcome only)*

*b. Adjusted for readmission hospital teaching status, readmission hospital urban/rural status, readmission hospital ownership*

*c. Models a + b covariates included*

**Appendix Table 4: Analysis of COVID-19 Admissions and COVID-19 Readmissions, National Readmissions Database, March-November 2020**

|  | Unadjusted | Clinical/Demo^a^ | Hospital^b^ | Full^c^ |
| --- | --- | --- | --- | --- |
| Died during Readmission (OR, 95% CI) | 1.34 (1.25, 1.43) | 1.27 (1.18, 1.36) | 1.33 (1.25, 1.43) | 1.25 (1.16, 1.35) |
| LOS readmission (Regression coefficient, 95% CI) | 1.59 (1.36, 1.82) | 1.32 (1.10, 1.55) | 1.60 (1.36, 1.83) | 1.31 (1.09, 1.54) |

*Reference group is nonfragmented readmissions.*

*a. Adjusted for sex, age, zip income quartile, insurance payer, resident of the state the readmission occurred in, APRDRG risk of mortality, Elixhauser Comorbidity Score, LOS of admission (LOS outcome only)*

*b. Adjusted for readmission hospital teaching status, readmission hospital urban/rural status, readmission hospital ownership*

*c. Models a + b covariates included*

**Appendix Table 5: Analysis of Readmissions for a Primary Diagnosis of COVID-19, National Readmissions Database, March-November 2020**

|  |  | Unadjusted | Clinical/Demo^a^ | Hospital^b^ | Full^c^ |
| --- | --- | --- | --- | --- | --- |
| Died during readmission | **Non-COVID-19 Readmissions** | 1.25 (1.21, 1.29) | 1.19 (1.15, 1.23) | 1.25 (1.21, 1.29) | 1.18 (1.15, 1.22) |
|  | **COVID-19 Primary Diagnosis of Readmission** | 1.12 (1.05. 1.20) | 1.13 (1.05, 1.21) | 1.13 (1.06, 1.20) | 1.12 (1.05, 1.20) |
| LOS readmission | **Non-COVID-19 Readmissions** | 1.00 (0.92, 1.09) | 0.89 (0.80, 0.97) | 1.03 (0.95, 1.11) | 0.90 (0.82, 0.99) |
|  | **COVID-19 Primary Diagnosis of Readmission** | 0.96 (0.71, 1.21) | 0.89 (0.63, 1.13) | 0.96 (0.70, 1.21) | 0.85 (0.60, 1.10) |

*Reference group is nonfragmented readmissions. 2020 COVID and non-COVID analyses are for March-December only.*

*a. Adjusted for sex, age, zip income quartile, insurance payer, resident of the state the readmission occurred in, APRDRG risk of mortality, Elixhauser Comorbidity Score, LOS of admission (LOS outcome only)*

*b. Adjusted for readmission hospital teaching status, readmission hospital urban/rural status, readmission hospital ownership*

*c. Models a + b covariates included*

**Appendix Table 6: Analysis of Admissions for a Primary Diagnosis of COVID-19, National Readmissions Database, March-November 2020**

|  |  | Unadjusted | Clinical/Demo^a^ | Hospital^b^ | Full^c^ |
| --- | --- | --- | --- | --- | --- |
| Died during readmission | **Non-COVID-19 Admissions** | 1.24 (1.21, 1.28) | 1.21 (1.17, 1.25) | 1.24 (1.21, 1.28) | 1.20 (1.17, 1.24) |
|  | **COVID-19 Primary Diagnosis of Admission** | 1.27 (1.18, 1.37) | 1.21 (1.12, 1.31) | 1.26 (1.17, 1.36) | 1.18 (1.09, 1.28) |
| LOS readmission | **Non-COVID-19 Admissions** | 1.00 90.91, 1.08) | 0.87 (0.79, 0.95) | 1.01 (0.93, 1.09) | 0.87 (0.79, 0.94) |
|  | **COVID-19 Primary Diagnosis of Admission** | 1.50 (1.27, 1.74) | 1.23 (1.00, 1.45) | 1.49 (1.26, 1.73) | 1.22 (0.99, 1.45) |

*Reference group is nonfragmented readmissions.*

*a. Adjusted for sex, age, zip income quartile, insurance payer, resident of the state the readmission occurred in, APRDRG risk of mortality, Elixhauser Comorbidity Score, LOS of admission (LOS outcome only)*

*b. Adjusted for readmission hospital teaching status, readmission hospital urban/rural status, readmission hospital ownership*

*c. Models a + b covariates included*

**Appendix Table 7:** **Association between Fragmented Readmissions and the Length of Stay of the Readmission, National Readmissions Database, Limited to Patients who Survived their Readmission, March-November 2018, 2019, and 2020**

| *Regression Coefficients (95% CI)* | Unadjusted | Clinical/Demo^a^ | Hospital^b^ | Full^c^ |
| --- | --- | --- | --- | --- |
| 2018 | 0.83 (0.75, 0.90) | 0.82 (0.74, 0.89) | 0.85 (0.78, 0.93) | 0.83 (0.76, 0.91) |
| 2019 | 0.84 (0.76, 0.92) | 0.81 (0.73, 0.89) | 0.87 (0.79, 0.95) | 0.83 (0.74, 0.90) |
| 2020 COVID-19 Readmissions | 1.43 (1.21, 1.64) | 1.31 (1.09, 1.52) | 1.43 (1.22, 1.65) | 1.30 (1.09, 1.53) |
| 2020 Non-COVID-19 Readmissions | 0.95 (0.87, 1.03) | 0.89 (0.81, 0.97) | 0.98 (0.90, 1.06) | 0.90 (0.82, 0.98) |

*Reference group is nonfragmented readmissions. 2020 COVID and non-COVID analyses are for March-December only.*

*a. Adjusted for sex, age, zip income quartile, insurance payer, resident of the state the readmission occurred in, APRDRG risk of mortality, Elixhauser Comorbidity Score, LOS of admission (LOS outcome only)*

*b. Adjusted for readmission hospital teaching status, readmission hospital urban/rural status, readmission hospital ownership*

*c. Models a + b covariates included*

**Appendix Table 8: Association between 30-Day Fragmented Readmissions and the Odds of Readmission In-Hospital Mortality, Stratified by Number of Admission-Readmission Pairs, National Readmissions Database, March-November 2018, 2019, and 2020**

|  |  | Unadjusted | Clinical/Demo^a^ | Hospital^b^ | Full^c^ |
| --- | --- | --- | --- | --- | --- |
| 2018 | 1 admission | 1.32 (1.28, 1.36) | 1.21 (1.16, 1.25) | 1.32 (1.28, 1.36) | 1.20 (1.15, 1.24) |
|  | 2 admission-readmission pairs | 1.18 (1.13, 1.23) | 1.17 (1.12, 1.22) | 1.18 (1.14, 1.23) | 1.16 (1.11, 1.21) |
|  | >2 admission-readmission pairs | 1.04 (1.00, 1.09) | 1.14 (1.09, 1.19) | 1.06 (1.02, 1.10) | 1.15 (1.10, 1.20) |
| 2019 | 1 admission | 1.34 (1.29, 1.39) | 1.23 (1.18, 1.28) | 1.34 (1.30, 1.39) | 1.22 (1.17, 1.27) |
|  | 2 admission-readmission pairs | 1.19 (1.14, 1.24) | 1.17 (1.12, 1.22) | 1.20 (1.15, 1.25) | 1.17 (1.12, 1.22) |
|  | >2 admission-readmission pairs | 1.06 (1.02, 1.10) | 1.14 (1.10, 1.19) | 1.08 (1.04, 1.12) | 1.15 (1.10, 1.20) |
| 2020 COVID-19 Readmissions | 1 admission | 1.30 (1.23, 1.38) | 1.21 (1.13, 1.29) | 1.29 (1.22, 1.37) | 1.20 (1.12, 1.28) |
|  | 2 admission-readmission pairs | 1.18 (1.09, 1.27) | 1.15 (1.06, 1.25) | 1.17 (1.08, 1.26) | 1.14 (1.05, 1.24) |
|  | >2 admission-readmission pairs | 1.05 (0.97, 1.14) | 1.11 (1.01, 1.21) | 1.05 (0.97, 1.14) | 1.10 (1.01, 1.21) |
| 2020 Non-COVID-19 Readmissions | 1 admission | 1.36 (1.32, 1.40) | 1.15 (1.11, 1.20) | 1.36 (1.32, 1.40) | 1.15 (1.11, 1.19) |
|  | 2 admission-readmission pairs | 1.21 (1.16, 1.26) | 1.12 (1.07, 1.17) | 1.22 (1.17, 1.27) | 1.12 (1.07, 1.16) |
|  | >2 admission-readmission pairs | 1.12 (1.07, 1.17) | 1.16 (1.11, 1.21) | 1.13 (1.08, 1.18) | 1.16 (1.11, 1.21) |

*Reference group is nonfragmented readmissions*

*a. Adjusted for sex, age, zip income quartile, insurance payer, resident of the state the readmission occurred in, APRDRG risk of mortality, Elixhauser Comorbidity Score, LOS of admission (LOS outcome only)*

*b. Adjusted for readmission hospital teaching status, readmission hospital urban/rural status, readmission hospital ownership*

*c. Models a + b covariates included*

**Appendix Table 9: Association between 30-Day Fragmented Readmissions and Readmission LOS, Stratified by Number of Admission-Readmission Pairs, National Readmissions Database, March-November 2018, 2019, and 2020**

|  |  | Unadjusted | Clinical/Demo^a^ | Hospital^b^ | Full^c^ |
| --- | --- | --- | --- | --- | --- |
| 2018 | 1 admission | 1.10 (1.02, 1.19) | 0.90 (0.81, 0.98) | 1.13 (1.05, 1.22) | 0.91 (0.83, 1.00) |
|  | 2 admission-readmission pairs | 0.95 (0.86, 1.05) | 0.89 (0.79, 0.98) | 0.99 (0.89, 1.08) | 0.90 (0.80, 0.99) |
|  | >2 admission-readmission pairs | 0.63 (0.54, 0.71) | 0.69 (0.61, 0.78) | 0.65 (0.57, 0.73) | 0.70 (0.61, 0.78) |
| 2019 | 1 admission | 1.11 (1.02, 1.20) | 0.90 (0.81, 0.99) | 1.15 (1.06, 1.24) | 0.92 (0.83, 1.01) |
|  | 2 admission-readmission pairs | 0.98 (0.88, 1.09) | 0.88 (0.78, 0.99) | 1.02 (0.91, 1.12) | 0.90 (0.80, 1.00) |
|  | >2 admission-readmission pairs | 0.65 (0.55, 0.74) | 0.69 (0.60, 0.78) | 0.67 (0.58, 0.76) | 0.70 (0.61, 0.79) |
| 2020 COVID-19 Readmissions | 1 admission | 1.24 (0.96, 1.51) | 0.95 (0.68, 1.22) | 1.23 (0.95, 1.51) | 0.93 (0.65, 1.20) |
|  | 2 admission-readmission pairs | 1.11 (0.76, 1.45) | 0.93 (0.60, 1.27) | 1.11 (0.76, 1.45) | 0.92 (0.59, 1.26) |
|  | >2 admission-readmission pairs | 0.95 (0.63, 1.26) | 0.94 (0.63, 1.25) | 0.92 (0.61, 1.24) | 0.92 (0.61, 1.23) |
| 2020 Non-COVID-19 Readmissions | 1 admission | 1.22 (1.13, 1.31) | 0.95 (0.85, 1.04) | 1.24 (1.15, 1.33) | 0.96 (0.87, 1.05) |
|  | 2 admission-readmission pairs | 1.07 (0.96, 1.17) | 0.92 (0.82, 1.03) | 1.10 (0.99, 1.20) | 0.94 (0.83, 1.04) |
|  | >2 admission-readmission pairs | 0.68 (0.59, 0.77) | 0.71 (0.62, 0.79) | 0.70 (0.62, 0.79) | 0.72 (0.63, 0.80) |

*Reference group is nonfragmented readmissions*

*a. Adjusted for sex, age, zip income quartile, insurance payer, resident of the state the readmission occurred in, APRDRG risk of mortality, Elixhauser Comorbidity Score, LOS of admission (LOS outcome only)*

*b. Adjusted for readmission hospital teaching status, readmission hospital urban/rural status, readmission hospital ownership*

*c. Models a + b covariates included*

**Appendix Table 10: Association between 90-Day Fragmented Readmissions and the Odds of Readmission In-Hospital Mortality, National Readmissions Database, March-November 2018, 2019, and 2020**

|  | Unadjusted | Clinical/Demo^a^ | Hospital^b^ | Full^c^ |
| --- | --- | --- | --- | --- |
| 2018 | 1.13 (1.10, 1.17) | 1.19 (1.15, 1.22) | 1.14 (1.11, 1.18) | 1.19 (1.15, 1.22) |
| 2019 | 1.16 (1.13, 1.19) | 1.20 (1.16, 1.23) | 1.17 (1.14, 1.20) | 1.20 (1.17, 1.23) |
| 2020 COVID-19 Readmissions | 1.16 (1.11, 1.21) | 1.15 (1.10, 1.20) | 1.15 (1.11, 1.20) | 1.15 (1.10, 1.20) |
| 2020 Non-COVID-19 Readmissions | 1.20 (1.17, 1.24) | 1.17 (1.14, 1.21) | 1.21 (1.18, 1.24) | 1.17 (1.14, 1.21) |

*Reference group is nonfragmented readmissions*

*a. Adjusted for sex, age, zip income quartile, insurance payer, resident of the state the readmission occurred in, APRDRG risk of mortality, Elixhauser Comorbidity Score, LOS of admission (LOS outcome only)*

*b. Adjusted for readmission hospital teaching status, readmission hospital urban/rural status, readmission hospital ownership*

*c. Models a + b covariates included*

**Appendix Table 11: Association between 90-Day Fragmented Readmissions and the Length of Stay of the Readmission, National Readmissions Database, March-November 2018, 2019, and 2020**

|  | Unadjusted | Clinical/Demo | Hospital | Full |
| --- | --- | --- | --- | --- |
| 2018 | 0.73 (0.66, 0.80) | 0.72 (0.64, 0.79) | 0.74 (0.67, 0.81) | 0.72 (0.65, 0.79) |
| 2019 | 0.74 (0.66, 0.81) | 0.71 (0.63, 0.78) | 0.76 (0.68, 0.83) | 0.71 (0.64, 0.79) |
| 2020 COVID-19 Readmissions | 1.02 (0.85, 1.20) | 0.88 (0.71, 1.05) | 1.01 (0.83, 1.18) | 0.85 (0.68, 1.02) |
| 2020 Non-COVID-19 Readmissions | 0.81 (0.74, 0.89) | 0.75 (0.68, 0.83) | 0.84 (0.76, 0.91) | 0.76 (0.69, 0.84) |

*Reference group is nonfragmented readmissions*

*a. Adjusted for sex, age, zip income quartile, insurance payer, resident of the state the readmission occurred in, APRDRG risk of mortality, Elixhauser Comorbidity Score, LOS of admission (LOS outcome only)*

*b. Adjusted for readmission hospital teaching status, readmission hospital urban/rural status, readmission hospital ownership*

*c. Models a + b covariates included*
